# The impact of different imputation methods on estimates and model performance: an example using a risk prediction model for premature mortality

**DOI:** 10.1186/s12963-024-00331-3

**Published:** 2024-06-17

**Authors:** Mackenzie Hurst, Meghan O’Neill, Lief Pagalan, Lori M. Diemert, Laura C. Rosella

**Affiliations:** 1 Population Health Analytics Lab, Dalla Lana School of Public Health, University of Toronto, Toronto, ON Canada; 2 ICES, Toronto, ON Canada; 3 Schwartz Reisman Institute for Technology and Society, University of Toronto, Toronto, ON Canada; 4 Laboratory Medicine and Pathobiology, Temerty Faculty of Medicine, University of Toronto, Toronto, ON Canada; 5 Institute for Better Health, Trillium Health Partners, Mississauga, ON Canada

**Keywords:** Prediction model, Missing data, Imputation methods, Prediction models, Perforamance measures, Population health

## Abstract

**Objective:**

To compare how different imputation methods affect the estimates and performance of a prediction model for premature mortality.

**Study Design and Setting:**

Sex-specific Weibull accelerated failure time survival models were run on four separate datasets using complete case, mode, single and multiple imputation to impute missing values. Six performance measures were compared to access predictive accuracy (Nagelkerke R^2^, integrated brier score), discrimination (Harrell’s c-index, discrimination slope) and calibration (calibration in the large, calibration slope).

**Results:**

The highest proportion of missingness for a single variable was 10.86% for the female model and 8.24% for the male model. Comparing the performance measures for complete case, mode, single and multiple imputation: the Nagelkerke R^2^ values for the female model was 0.1084, 0.1116, 0.1120 and 0.111–0.1120 with the male model exhibited similar variation of 0.1050, 0.1078, 0.1078 and 0.1078–0.1081. Harrell’s c-index also demonstrated small variation with values of 0.8666, 0.8719, 0.8719 and 0.8711–0.8719 for the female model and 0.8549, 0.8548, 0.8550 and 0.8550–0.8553 for the male model.

**Conclusion:**

In the scenarios examined in this study, mode imputation performed well when using a population health survey compared to single and multiple imputation when predictive performance measures is the main model goal. To generate unbiased hazard ratios, multiple imputation methods were superior. This study shows the need to consider the best imputation approach for a predictive model development given the conditions of missing data and the goals of the analysis.

**Supplementary Information:**

The online version contains supplementary material available at 10.1186/s12963-024-00331-3.

## Introduction

Missing data is an inevitable challenge encountered in health surveys, which can compromise the representativeness of the sample, introduce bias, and reduce statistical power [1]. Several factors contribute to missing data, including non-response, and survey administration errors. To address this issue, imputation methods have been developed, with several techniques employed in practice [2]. The choice of imputation method depends on several factors, including the type and pattern of missing data, the assumptions about the missingness mechanism, and the specific goals of the analysis [1, 2].

Prediction models are valuable tools that estimate the likelihood of future outcomes or events based on available data. These models serve diverse purposes in healthcare, clinical care and population health. Clinical risk prediction models assess individual patient risk and support treatment decisions, often relying on data from electronic patient records (e.g., blood pressure, bloodwork, genetic markers) [3]. On the other hand, population risk algorithms predict disease incidence, evaluate the impact of risk factors, and inform population health interventions directed at groups of people versus at the individual level [4]. The accuracy and reliability of a prediction model largely depends on the quality and representativeness of the data, which can be influenced by the presence of missing data and the methods used to address it [5]. Existing prediction model reporting guidelines, such as the transparent reporting of a multivariable prediction model for individual prognosis or diagnosis (TRIPOD), recommend reporting on missingness in development and validation datasets and how missing data were addressed [6]. Despite these recommendations, the reporting and handling of missing data in prediction models is often inadequate [7–9].

Although there is existing literature on imputation methods in the context of survey data, there is a notable gap in our understanding regarding the impact of missing data for prediction models based on population surveys. Therefore, the objective of this study is to compare four common imputation methods, including complete case, mode imputation, single imputation, and multiple imputation, for handling missing values. This comparison aims to assess the effects of each imputation technique on model estimates and evaluate their impact on model performance.

## Methods

The Premature Mortality Population Risk Tool (PreMPoRT) [10, 11] was developed and validated to predict the five-year incidence of premature mortality among Canadian adults. Model predictors included sociodemographic characteristics, self-perceived measures, health behaviours, and chronic conditions from national survey data. PreMPoRT demonstrated strong reproducibility and transportability in different validation data and performed well among important equity-stratified subgroups. Additional details about the development and validation of PreMPoRT are found elsewhere [10, 11].

We apply four missing data approaches: complete case, mode imputation, single imputation and multiple imputation using fully conditional specification (FCS) [12]. Six performance measures were used to assess the impact of each imputation method on the prediction model. The study received ethics approval from the University of Toronto Research Ethics Board (Protocol #37499). This work was supported by the Canadian Institutes of Health Research Operating Grants (FRNs: 72056684 and 72051628). Laura Rosella is also supported by a Canada Research Chair in Population Health Analytics (FRN: 72060091).

### Data sources

PreMPoRT used data from the Canadian Community Health Survey (CCHS), a cross-sectional survey containing information on self-reported sociodemographic characteristics, health status, health care utilization, and health determinants. The surveyed population represents 98% of the Canadian population aged 12 and older [13] and uses a complex-survey design, including clustering and stratification, to represent all regions in Canada. The CCHS was linked to the Canadian Vital Statistics Database (CVSD) to ascertain premature mortality during a five-year follow-up period after CCHS interview date [14]. Data were held at the Statistics Canada Research Data Centre.

### Participants

The study cohort consisted of participants who contributed to any of the first six cycles, 1.1 (2000/01), 2.1 (2003/04), 3.1 (2005/06), 2007/08, 2009/10 or 2011/12 of the CCHS. Individuals were removed from the cohort if they were pregnant or living in the Territories (Nunavut, Northwest Territories and Yukon) at the time of their CCHS interview date. Since PreMPoRT was developed for the Canadian adult population, individuals under 18 years old or over 75 were excluded.

### Model specification

PreMPoRT predicts premature mortality, which under the Canadian Institute of Health Information (CIHI), is defined as any death under the age of 75 [15]. Using death dates from the CVSD, the outcome is all-cause mortality within five years after CCHS interview date or the participant’s 75th birthday. PreMPoRT was developed using sex-specific Weibull accelerated failure time models. Participants were followed for five years after interview date, death or until 75 years old, whichever came first.

Using 38 candidate predictors [10], PreMPoRT identified 12 predictors for the female model and 13 predictors for the male model. Both models contained age, household income quintile, education level, self-perceived general health, cigarette smoking, emphysema/COPD, heart disease, diabetes, cancer, and stroke. Body-mass-index (BMI) and physical activity were unique to the female model with marital status, Alzheimer’s disease, and arthritis being unique to the male model.

To accurately represent the Canadian population, CCHS survey weights were developed by Statistics Canada to handle the complex-survey design and to represent certain demographic groups properly [16]. Since multiple cycles were used in the analysis, CCHS survey weights were pooled and divided by the number of cycles [17].

### Imputing missing values: four approaches

We used four different missing data methods to impute missing values. The first method was complete case, where any participant that had any missing predictor(s) was removed from the analysis. The second was mode imputation, where within each sex-stratified CCHS cycle, the most common value for any predictor(s) was imputed as the missing value.

The third method was single imputation using FCS [12]. Although PreMPoRT identified 12 predictors for females and 13 for males, imputation was run using all 38 candidate variables and the outcome [18]. Imputation was run separately for each cycle with the addition of stratifying by sex. However, due to converge issues all chronic conditions that had a low prevalence and less than 1% missingness were imputed as the absence of the condition (i.e., mode imputation for variables with less than 1% missing). These chronic conditions included emphysema/COPD, heart disease, diabetes, cancer, stroke, Alzheimer’s disease and arthritis. Afterwards, FCS was run five times as burn-in iterations to find convergence of the imputed values to create the imputed dataset. FCS used different regression models for each variable type, including logistic regression for binary variables, discriminant function for nominal variables and ordinal logistic regression for ordinal variables with more than two categories. Each variable was imputed within each CCHS cycle with the exception of anxiety and mood disorder in the first cycle as these questions were not asked in that cycle. After imputing all other variables within each cycle, anxiety and mood disorder for the first cycle were imputed using the next two CCHS cycles, as these were the other cycles within the development dataset. When building a prediction model it is important to avoid leakage between development and validation sets, as such imputing within each CCHS cycle as well as imputing anxiety and mood disorder within just the development cycles avoids all leakage from imputation.

The final method was multiple imputation (MI) which applied the same approach as single imputation to create four additional datasets for a total of five. The goal of MI is to generate multiple imputed datasets to observe how the distribution of the imputed values affects the results of the model.

### Model performance and measures

To compare the effects of the imputation methods on the prediction model, the Weibull specific model parameters, hazard ratios (HRs), and performance measures were compared. The Weibull model parameters include the scale and shape parameters as well as the intercept. The hazard ratios compare the proportional increase in the rate of premature mortality versus the reference group and were calculated for each predictor in the model. Finally, six performance measures were compared to assess the model’s overall predictive accuracy, discrimination, and calibration.

The Nagelkerke R^2^ and Integrated Brier Score were used to assess the predictive accuracy. The Nagelkerke R^2^ measures the percent of variance explained by the model with a target value of one. The Integrated Brier Score measures the average squared difference between the outcome and the predicted risk (while taking censoring into account) with a target value of zero.

Discrimination is how well the model can differentiate between those who experience an outcome versus those who did not. This was assessed using Harrell’s concordance index (c-index) which is the fraction of the number of concordant pairs over the number of concordant pairs and discordant pairs [18]. A pair compares two participants in the study, and if the individual who had an event first had a higher predicted risk (concordant pair) the model properly predicted the outcome. However, if that individual had a lower predicted risk (discordant pair) then the model did not properly predict the outcome. Discrimination will also be assessed using time-specific discrimination slope, which is the difference in the average predicted risk of those who had an event and those who did not have an outcome.

Finally, calibration will be measured in the large and calibration slope. Calibration in the large is the difference between the average observed risk (normally calculated using Kaplan-Meier curves) and the average predicted risk. The calibration slope assesses if the betas are well-calibrated for the model. A slope of one indicates perfect calibration, less than one indicates the betas are overestimating the predicted risk, and more than one indicates the betas are underestimating the predicted risk. In addition, calibration plots were produced to show further the effect of imputation methods on the calibration of the prediction model.

## Results

The highest proportion of missingness in any one variable was 10.86% for the female model and 8.24% for the male model. All chronic conditions, marital status, self-perceived general health and physical activity had less than 1% missingness. BMI, smoking status and individual education all had between 1% and 5% missingness, with income quintiles being the only variable with more than 5% missingness.

### Baseline characteristics

Table 1 shows the weighted percent of baseline characteristics with unweighted total counts from the cohort rounded to the nearest thousand to adhere to Statistics Canada’s export requirements. All datasets have a total of 267,000 for females and 233,000 for males, except for the complete case, which had a total of 221,000 (17% removed) for females and 195,000 (16% removed) for males. Across all imputations, a total of 1.41% females and 2.06% of males experienced premature death, except for complete case (1.27% premature deaths for females and 1.93% premature deaths for males). There were no notable differences across imputation methods, apart from household income quintiles, which had a missingness of 10.86% for females and 8.24% for males. The lowest income quintile for females had a missingness of 15.11% for complete case and 15.50 − 21.14% for the remaining imputation methods. For males, the biggest difference was in the highest income quintile, with 29.24% missingness for complete case and 28.48 − 31.92% for other imputation methods.

**Table 1:** Baseline characteristics

Characteristic	Original dataset	Complete case	Mode imputation	Single imputation	Multiple imputation [min - max]^1^
**FEMALE**	**(n = 267,000)**	**n=(221,000)**	**(n = 267,000)**	**(n = 267,000)**	**(n = 267,000)**
**Premature deaths (%)**	1.4	1.3	1.4	1.4	1.4
**Follow-up time**, mean (SD), years	4.9 (4.5)	4.9 (4.2)	4.9 (4.5)	4.9 (4.5)	4.9 (4.5)
**Age Group (%)**					
Missing	0.0	-	-	-	-
18–24	12.7	11.4	12.7	12.7	[12.7–12.7]
25–34	17.4	18.4	17.4	17.4	[17.4–17.4]
35–44	20.6	22.3	20.6	20.6	[20.6–20.6]
45–54	21.3	21.5	21.3	21.3	[21.3–21.3]
55–64	17.0	16.1	17.0	17.0	[17.0–17.0]
65–74	11.1	10.3	11.1	11.1	[11.1–11.1]
**Household income quintile**					
Missing	10.9	-	-	-	-
Q1 (lowest)	13.8	15.1	21.1	15.5	[15.5–15.6]
Q2	14.5	16.2	14.5	16.4	[16.3–16.5]
Q3	18.5	20.8	18.5	20.8	[20.8–20.8]
Q4	22.0	24.9	25.6	24.7	[24.6–24.8]
Q5 (highest)	20.3	23.0	20.3	22.5	[22.5–22.6]
**Individual education**					
Missing	4.5	-	-	-	-
Less than secondary school graduation	7.1	7.2	7.1	7.6	[7.6–7.6]
Secondary school graduation	10.5	10.8	10.5	11.1	[11.1–11.2]
Post-secondary education (complete and partial)	77.9	82.0	82.4	81.4	[81.3–81.4]
**Marital status**					
Missing	0.1	-	-	-	-
Single never married	22.1	20.6	22.1	22.1	[22.1–22.1]
Domestic partner (married/common law)	63.6	64.8	63.7	63.7	[63.6–63.7]
Widowed/separated/divorced	14.2	14.6	14.2	14.2	[14.2–14.2]
**BMI categories**					
Missing	3.4	-	-	-	-
< 18.5	3.9	3.8	3.9	4.0	[4.0–4.0]
18.5 to < 25.0	51.1	52.6	54.5	52.7	[52.7–52.7]
25.0 to < 30.0	25.8	26.9	25.8	26.8	[26.8–26.8]
30.0 to < 35.0	10.5	11.1	10.5	10.9	[10.9–11.0]
35.0 to < 40.0	3.5	3.7	3.5	3.6	[3.6–3.6]
≥ 40.0	1.8	1.9	1.8	1.9	[1.9–1.9]
**Self-perceived general health**					
Missing	0.1	-	-	-	-
Poor	2.7	2.5	2.7	2.7	[2.7–2.7]
Fair	8.6	8.2	8.6	8.6	[8.6–8.6]
Good	28.3	27.5	28.3	28.3	[28.3–28.3]
Very good	38.0	38.8	38.1	38.0	[38.0–38.0]
Excellent	22.4	23.0	22.4	22.4	[22.4–22.4]
**Physical activity** ^**2**^					
Missing	1.6	-	-	-	-
Active	22.4	22.7	22.4	22.8	[22.7–22.8]
Moderately active	25.4	26.2	25.4	25.7	[25.7–25.8]
Inactive	50.6	51.2	52.2	51.5	[51.5–51.5]
**Smoking status**					
Missing	3.1	-	-	-	-
Never smoker	54.0	54.7	57.1	55.7	[55.7–55.7]
Former light smoker	16.6	17.7	16.6	17.1	[17.1–17.2]
Former heavy smoker	5.0	5.3	5.0	5.2	[5.2–5.2]
Current light smoker	18.4	19.3	18.4	19.0	[19.0–19.0]
Current heavy smoker	2.9	3.0	2.9	3.0	[3.0–3.0]
**Self-reported chronic conditions**					
**Emphysema/COPD**					
Missing	0.1	-	-	-	-
Yes	1.7	1.7	1.7	1.7	[1.7–1.7]
**Heart disease**					
Missing	0.1	-	-	-	-
Yes	3.3	3.2	3.3	3.3	[3.3–3.3]
**Diabetes**					
Missing	0.1	-	-	-	-
Yes	4.7	4.5	4.7	4.7	[4.7–4.7]
**Cancer**					
Missing	0.1	-	-	-	-
Yes	1.8	1.8	1.8	1.8	[1.8–1.8]
**Stroke**					
Missing	0.0	-	-	-	-
Yes	0.8	0.7	0.8	0.8	[0.8–0.8]
**MALE**	(*n* = 233,000)	n=(195,000)	(*n* = 233,000)	(*n* = 233,000)	(*n* = 233,000)
**Premature deaths (%)**	2.1	1.9	2.1	2.1	[2.1–2.1]
**Follow-up time**, mean (SD), years					
**Age Group**					
Missing	0.0	-	-	-	-
18–24	13.6	11.9	13.6	13.6	[13.6–13.6]
25–34	18.5	19.0	18.5	18.5	[18.5–18.5]
35–44	20.9	22.5	20.9	20.9	[20.9–20.9]
45–54	20.5	21.0	20.5	20.5	[20.5–20.5]
55–64	16.8	16.4	16.8	16.8	[16.8–16.8]
65–74	9.9	9.3	9.9	9.9	[9.9–9.9]
**Household income quintile**					
Missing	8.2	-	-	-	-
Q1 (lowest)	10.6	11.2	10.6	11.6	[11.6–11.7]
Q2	12.4	13.3	12.4	13.6	[13.6–13.7]
Q3	17.8	19.3	17.8	19.5	[19.4–19.5]
Q4	24.5	26.9	27.2	26.7	[26.7–26.8]
Q5 (highest)	26.4	29.2	31.9	28.6	[28.5–28.6]
**Individual education**					
Missing	6.0	-	-	-	-
Less than secondary school graduation	6.2	6.4	6.2	6.8	[6.8–6.8]
Secondary school graduation	10.3	10.8	10.3	11.1	[11.1–11.2]
Post-secondary education (complete and partial)	77.4	82.8	83.5	82.1	[82.1–82.1]
**Marital status**					
Missing	0.1	-	-	-	-
Single never married	26.8	24.3	26.8	26.8	[26.8–26.8]
Domestic partner (married/common law)	65.7	68.0	65.8	65.8	[65.7–65.8]
Widowed/separated/divorced	7.4	7.7	7.4	7.4	[7.4–7.5]
**BMI categories**					
Missing	1.5	-	-	-	-
< 18.5	1.1	1.0	1.1	1.1	[1.1–1.1]
18.5 to < 25.0	39.6	39.5	41.0	40.1	[40.1–40.2]
25.0 to < 30.0	40.1	41.2	40.1	40.7	[40.6–40.7]
30.0 to < 35.0	13.6	14.0	13.6	13.8	[13.8–13.9]
35.0 to < 40.0	3.1	3.2	3.1	3.1	[3.1–3.1]
≥ 40.0	1.1	1.1	1.1	1.1	[1.1–1.1]
**Self-perceived general health**					
Missing	0.1	-	-	-	-
Poor	2.4	2.2	2.4	2.4	[2.4–2.4]
Fair	7.8	7.4	7.8	7.8	[7.8–7.8]
Good	28.4	28.2	28.4	28.5	[28.5–28.5]
Very good	37.9	38.8	37.9	37.9	[37.9–37.9]
Excellent	23.4	23.4	23.4	23.4	[23.4–23.4]
**Smoking status**					
Missing	3.5	-	-	-	-
Never smoker	44.4	45.6	47.9	46.0	[46.0–46.1]
Former light smoker	16.7	17.8	16.7	17.3	[17.3–17.3]
Former heavy smoker	9.4	9.9	9.4	9.8	[9.8–9.9]
Current light smoker	20.3	20.8	20.3	21.0	[20.9–21.0]
Current heavy smoker	5.7	5.8	5.7	5.9	[5.9–5.9]
**Self-reported chronic conditions**					
**Emphysema/COPD**					
Missing	0.1	-	-	-	-
Yes	1.5	1.5	1.5	1.5	[1.5–1.5]
**Heart disease**					
Missing	0.1	-	-	-	-
Yes	4.8	4.8	4.8	4.8	[4.8–4.8]
**Diabetes**					
Missing	0.1	-	-	-	-
Yes	5.8	5.7	5.8	5.8	[5.8–5.8]
**Cancer**					
Missing	0.1	-	-	-	-
Yes	1.5	1.5	1.5	1.5	[1.5–1.5]
**Stroke**					
Missing	0.0	-	-	-	-
Yes	0.9	0.8	0.9	0.9	[0.9–0.9]
**Arthitis**					
Missing	0.1	-	-	-	-
Yes	11.7	11.5	11.7	11.7	[11.7–11.7]
**Alzheimer’s**					
Missing	0.1	-	-	-	-
Yes	0.2	0.1	0.2	0.2	[0.2–0.2]

^1^Multiple Imputation created 5 datasets, as such this the range from the minimum percent within each dataset to the maximum percent in each dataset

^2^Physical activity was measured using average metabolic equivalent of task (MET) per day derived from a list of leisure-time physical activities (frequency and duration of activity)

### Performance measures

Table 2 shows the variation in performance measures when applying the four imputation methods. The Nagelkerke R^2^ for the female model was 0.1084 for complete case, with the remaining imputation methods ranging between 0.1111 and 0.1120. The Nagelkerke R^2^ for the male model was 0.1050 for complete case and a range of 0.1078–0.1081 for other imputation methods. The c-index results were as follows: complete case was 0.8666, for females and 0.8549 for male, with the remaining methods giving a range of 0.8711–0.8719 and 0.8548–0.8553 for females and males, respectively.

**Fig. 1:**
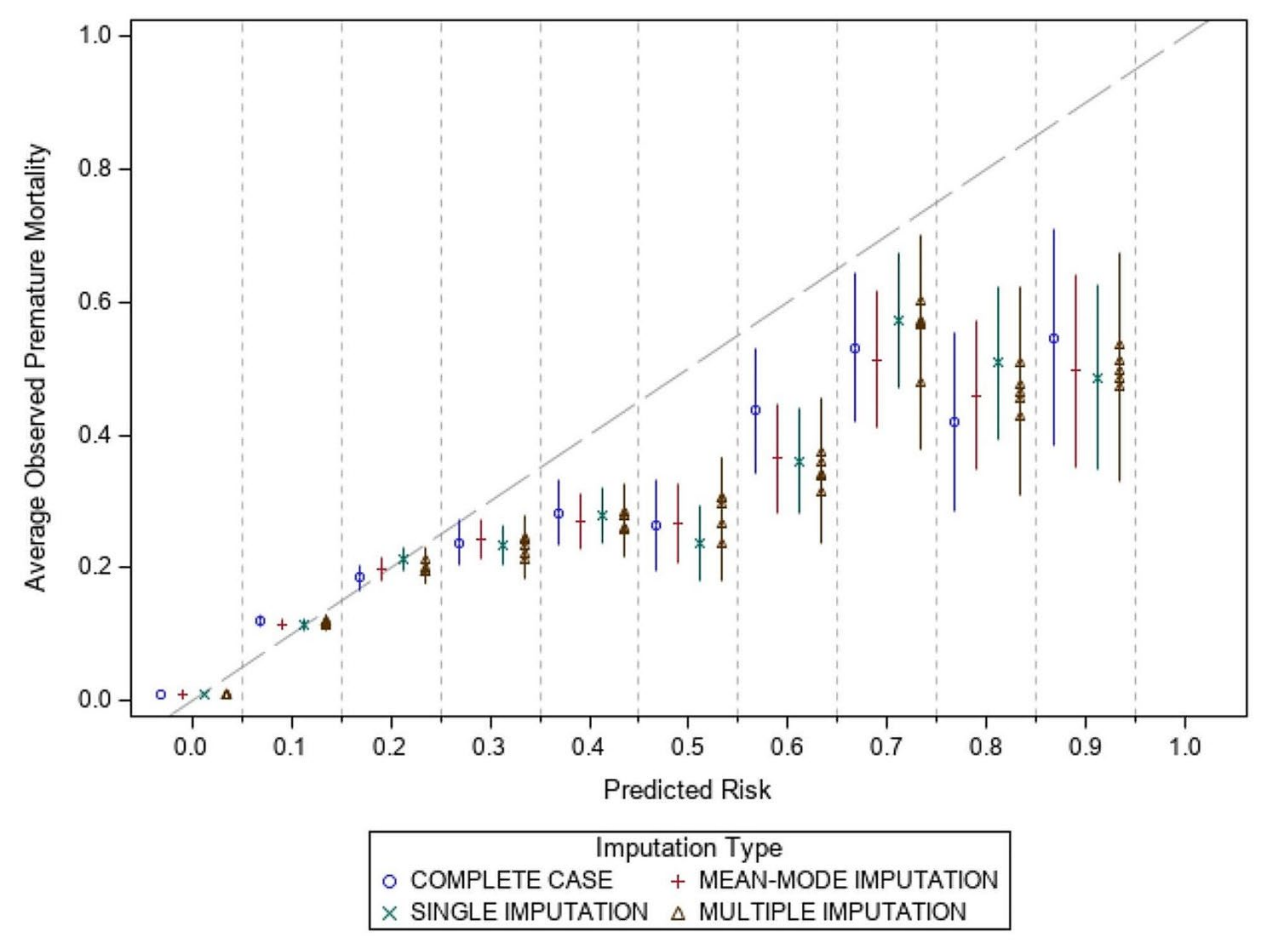
Calibration plot of predicted risk deciles versus average observed premature mortality for females

**Fig. 2:**
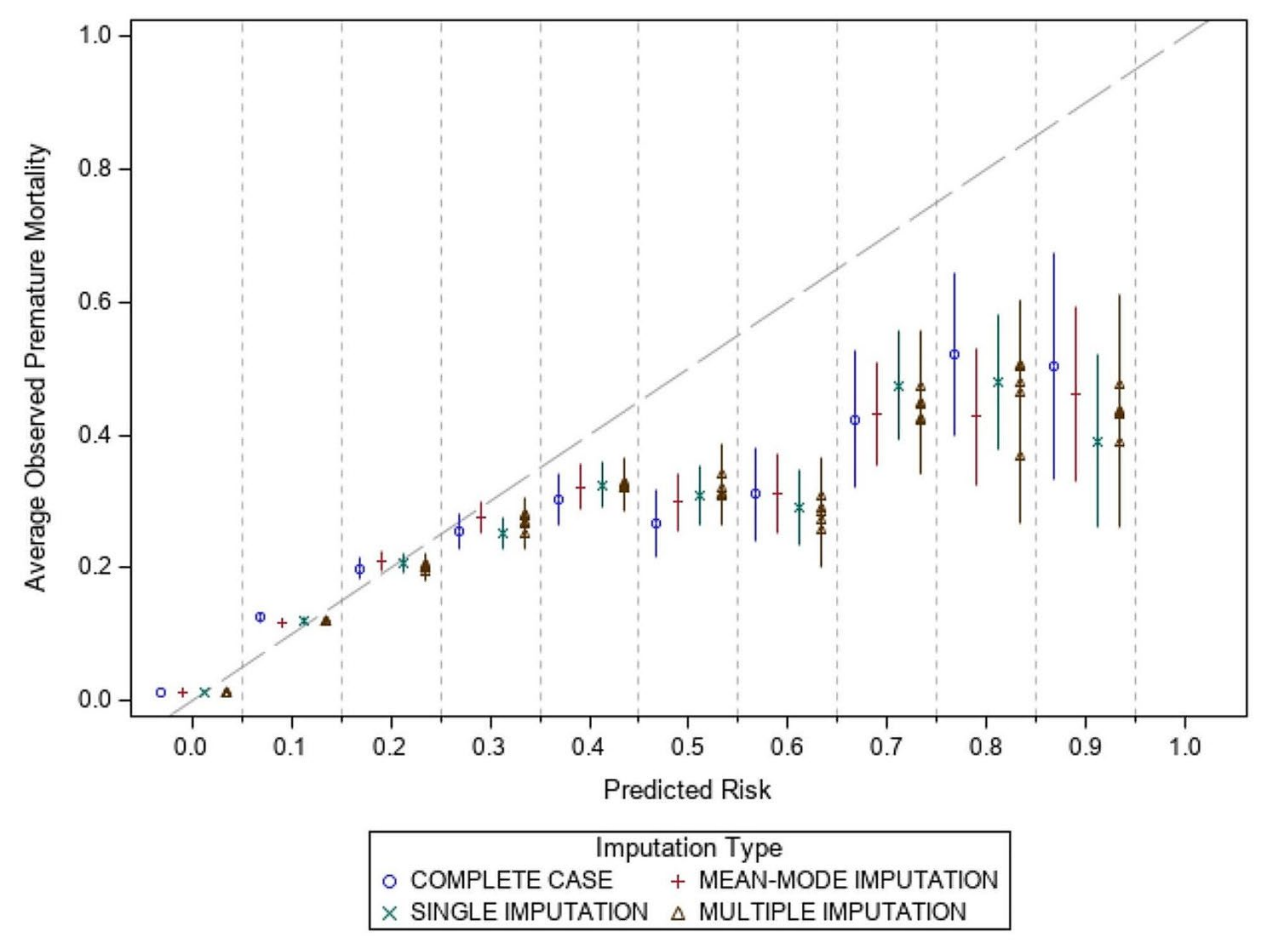
Calibration plot of predicted risk deciles versus average observed risk of premature mortality for males

The performance measures for calibration changed minimally across imputation methods. In addition to the performance measures, Figs. 1 and 2 show the average observed risk of premature mortality against the predicted risk of the model for females and males, respectively. Predicted risk is shown in deciles and the percent of observed cases that had a premature death in each decile was reported. Perfect calibration represents a slope of 1. The supplementary materials contain additional calibration plots from select predictors, including age groups, education level, ethnicity, immigration status and material deprivation. These show the percentage of premature deaths and compare them to the average predicted risk from each imputation method.

**Table 2:** Performance measures

	Complete	Mode	Single	Multiple
	Case	Imputation	Imputation	Imputation
FEMALE				
Nagelkerke R2^1^	0.1084	0.1116	0.1120	0.1111–0.1120
Integrated Brier Score^2^	0.0055	0.0063	0.0063	0.0063–0.0063
C-index^3^	0.8666	0.8719	0.8719	0.8711–0.8719
Discrimination Slope^4^	0.0892	0.0930	0.0936	0.0926–0.0938
Calibration in the large^5^	-0.0016	-0.0017	-0.0017	-0.0017, -0.0017
Calibration Slope^6^	1.0000	1.0000	1.0000	1.0000–1.0000
MALE				
Nagelkerke R2^1^	0.1050	0.1078	0.1078	0.1078–0.1081
Integrated Brier Score^2^	0.0086	0.0093	0.0093	0.0093–0.0093
C-index^3^	0.8549	0.8548	0.8550	0.8550–0.8553
Discrimination Slope^4^	0.0904	0.0988	0.0990	0.0990–0.0993
Calibration in the large^5^	-0.0020	-0.0022	-0.0022	-0.0022,-0.0022
Calibration Slope^6^	1.0000	1.0000	1.0000	1.0000–1.0000

^1^The Nagelkerke R2 measures the percent of variance explained by the model with a target value of one

^2^The Integrated Brier Score measures the average squared difference between the outcome and the predicted risk (while taking censoring into account) with a target value of zero

^3^Harrell’s concordance index (c-index) which is the fraction of the number of concordant pairs over the number of concordant pairs and discordant pairs

^4^Time-specific discrimination slope is the difference in the average predicted risk of those who had an event and those who did not have an outcome

^5^Calibration in the large is the difference between the average observed risk (normally calculated using Kaplan-Meier curves) and the average predicted risk

^6^The calibration slope assesses if the betas are well-calibrated for the model (with a slope of one indicating perfect calibration)

### Hazard ratios and confidence intervals

Table 3 shows the Weibull parameters and the HRs for the female and male models by imputation method. The female scale parameter was 0.7852 for complete case, and varied from 0.8194 to 0.8200 for the remaining imputation methods. The male scale parameter was 0.8137 for complete case and ranged from 0.8468 to 0.8472 for the other imputation methods. The HRs for all chronic conditions, age, self-perceived general health, cigarette smoking, physical activity, and marital status remained relatively unchanged between the imputation methods with the exception of complete case which did show noticeable differences across almost all predictors. Excluding complete case, household income demonstrated the biggest difference in confidence intervals for the female model. Specifically, the lowest income quintile (Q1) ranged from 1.22 to 1.26 for mode imputation to 1.09–1.33 for multiple imputation. The second highest quintile (Q4) ranged from 1.11 to 1.15 for mode imputation to 0.95–1.16 for multiple imputation. We observed similar variation for the male model with the lowest income quintile (Q1) ranging from 1.36 to 1.40 for mode imputation to 1.31–1.54 for multiple imputation and, the second highest quintile (Q4) ranged from 1.12 to 1.15 for mode imputation to 1.10–1.19 for multiple imputation.

**Table 3:** Hazard ratios

	Complete	Mode	Single	Multiple
	Case	Imputation	Imputation	Imputation
Variable	HR (95% CI)	HR (95% CI)	HR (95% CI)	HR (95% CI)
**FEMALE**				
**Weibull parameters**				
Scale parameter	0.7852	0.8196	0.8194	0.8194-0.8200
Shape parameter	1.2736	1.2201	1.2204	1.2195–1.2204
Intercept	12.2157	12.3887	12.3880	12.3151–12.3880
**Age**	1.08 (1.08–1.08)	1.08 (1.08–1.08)	1.08 (1.08–1.08)	1.08 (1.08–1.08)
**Household income quintile**				
Q1 (lowest)	1.33 (1.30–1.36)	1.24 (1.22–1.26)	1.25 (1.23–1.27)	1.21 (1.09–1.33)
Q2	1.14 (1.11–1.16)	1.09 (1.07–1.11)	1.15 (1.13–1.17)	1.07 (0.92–1.24)
Q3	1.13 (1.11–1.15)	1.09 (1.07–1.11)	1.06 (1.04–1.08)	1.05 (0.92–1.19)
Q4	1.14 (1.11–1.16)	1.13 (1.11–1.15)	1.09 (1.07–1.11)	1.05 (0.95–1.16)
Q5 (highest)	REF	REF	REF	REF
**Education**				
Less than secondary school graduation	1.13 (1.12–1.15)	1.13 (1.11–1.14)	1.10 (1.08–1.11)	1.12 (1.07–1.17)
Secondary school graduation	1.01 (1.00–1.03)	1.03 (1.02–1.05)	1.01 (1.00–1.03)	1.03 (0.99–1.07)
Post-secondary education (complete and partial)	REF	REF	REF	REF
**Body mass index (BMI) (kg/m** ^**2**^ **)**				
< 18.5	1.67 (1.62–1.71)	1.55 (1.52–1.58)	1.53 (1.49–1.56)	1.60 (1.45–1.77)
18.5 to < 25.0 (normal weight)	REF	REF	REF	REF
25.0 to < 30.0	0.82 (0.81–0.84)	0.74 (0.73–0.75)	0.74 (0.73–0.75)	0.78 (0.71–0.86)
30.0 to < 35.0	0.75 (0.73–0.76)	0.65 (0.64–0.66)	0.66 (0.65–0.67)	0.69 (0.64–0.74)
35.0 to < 40.0	0.82 (0.79–0.84)	0.67 (0.65–0.68)	0.69 (0.67–0.70)	0.71 (0.65–0.77)
≥ 40.0	1.03 (1.00–1.06)	0.91 (0.88–0.93)	0.85 (0.82–0.87)	0.89 (0.81–0.99)
**Self-perceived general health**				
Poor	5.29 (5.15–5.44)	6.20 (6.06–6.35)	6.34 (6.19–6.49)	6.29 (5.85–6.75)
Fair	2.42 (2.36–2.48)	2.83 (2.77–2.89)	2.84 (2.78–2.90)	2.83 (2.69–2.98)
Good	1.51 (1.47–1.54)	1.71 (1.68–1.75)	1.72 (1.68–1.75)	1.72 (1.63–1.80)
Very good	1.19 (1.17–1.22)	1.20 (1.18–1.23)	1.18 (1.16–1.21)	1.18 (1.13–1.23)
Excellent	REF	REF	REF	REF
**Cigarette smoking**				
Never smoked	REF	REF	REF	REF
Former light smoker	1.54 (1.52–1.57)	1.59 (1.57–1.61)	1.61 (1.59–1.63)	1.60 (1.57–1.63)
Former heavy smoker	2.03 (1.99–2.07)	1.93 (1.90–1.96)	1.97 (1.93–2.00)	1.96 (1.89–2.03)
Current light smoker	2.44 (2.40–2.48)	2.34 (2.31–2.37)	2.38 (2.34–2.41)	2.35 (2.27–2.44)
Current heavy smoker	2.90 (2.83–2.97)	2.76 (2.70–2.81)	2.76 (2.70–2.82)	2.77 (2.71–2.83)
**Physical activity**				
Inactive	1.30 (1.28–1.33)	1.40 (1.38–1.42)	1.39 (1.37–1.42)	1.36 (1.28–1.45)
Moderately active	1.02 (1.00–1.04)	1.09 (1.07–1.11)	1.11 (1.09–1.13)	1.08 (1.02–1.15)
Active	REF	REF	REF	REF
**Emphysema/COPD**	1.21 (1.19–1.24)	1.23 (1.20–1.25)	1.21 (1.19–1.23)	1.21 (1.19–1.24)
**Heart disease**	1.18 (1.16–1.21)	1.22 (1.20–1.23)	1.21 (1.19–1.22)	1.21 (1.19–1.23)
**Diabetes**	1.38 (1.36–1.41)	1.30 (1.29–1.32)	1.31 (1.29–1.33)	1.30 (1.27–1.33)
**Cancer**	5.75 (5.65–5.85)	5.31 (5.23–5.39)	5.30 (5.22–5.38)	5.27 (5.19–5.36)
**Stroke**	1.52 (1.48–1.56)	1.37 (1.34–1.40)	1.38 (1.35–1.42)	1.38 (1.35–1.42)
**MALE**				
**Weibull parameters**				
Scale parameter	0.8137	0.8472	0.8468	0.8468–0.8469
Shape parameter	1.2289	1.1804	1.1809	1.1808–1.1809
Intercept	11.6282	11.6657	11.7012	11.7010-11.7216
**Age**	1.08 (1.08–1.08)	1.08 (1.08–1.08)	1.08 (1.08–1.08)	1.08 (1.08–1.08)
**Household income quintile**				
Q1 (lowest)	1.61 (1.58–1.63)	1.38 (1.36–1.40)	1.39 (1.37–1.41)	1.42 (1.31–1.54)
Q2	1.65 (1.62–1.68)	1.46 (1.45–1.48)	1.46 (1.44–1.49)	1.51 (1.40–1.63)
Q3	1.40 (1.38–1.43)	1.23 (1.22–1.25)	1.27 (1.25–1.29)	1.30 (1.23–1.38)
Q4	1.22 (1.20–1.24)	1.14 (1.12–1.15)	1.12 (1.11–1.14)	1.14 (1.10–1.19)
Q5 (highest)	REF	REF	REF	REF
**Marital status**				
Domestic partner (married/common law)	0.60 (0.59–0.61)	0.56 (0.55–0.57)	0.57 (0.56–0.58)	0.57 (0.55–0.58)
Widowed/separated/divorced	1.01 (0.99–1.02)	0.90 (0.88–0.91)	0.91 (0.90–0.92)	0.90 (0.89–0.92)
Single never married	REF	REF	REF	REF
**Education**				
Less than secondary school graduation	1.12 (1.11–1.13)	1.10 (1.09–1.12)	1.15 (1.14–1.16)	1.10 (1.01–1.19)
Secondary school graduation	1.03 (1.02–1.04)	1.07 (1.05–1.08)	1.08 (1.06–1.09)	1.05 (0.99–1.12)
Post-secondary education (complete and partial)	REF	REF	REF	REF
**Self-perceived general health**				
Poor	4.61 (4.52–4.71)	5.48 (5.37–5.58)	5.41 (5.31–5.52)	5.37 (5.25–5.50)
Fair	2.24 (2.19–2.28)	2.49 (2.45–2.53)	2.47 (2.43–2.51)	2.47 (2.42–2.51)
Good	1.42 (1.39–1.44)	1.59 (1.57–1.62)	1.59 (1.56–1.61)	1.58 (1.56–1.61)
Very good	1.04 (1.02–1.06)	1.13 (1.11–1.15)	1.12 (1.10–1.14)	1.12 (1.10–1.14)
Excellent	REF	REF	REF	REF
**Cigarette smoking**				
Never smoked	REF	REF	REF	REF
Former light smoker	1.12 (1.10–1.13)	1.17 (1.15–1.18)	1.22 (1.20–1.23)	1.21 (1.16–1.26)
Former heavy smoker	1.65 (1.62–1.67)	1.56 (1.54–1.58)	1.60 (1.58–1.62)	1.61 (1.56–1.65)
Current light smoker	2.22 (2.19–2.26)	2.08 (2.06–2.11)	2.08 (2.05–2.10)	2.10 (2.04–2.17)
Current heavy smoker	3.09 (3.04–3.14)	2.87 (2.83–2.91)	2.88 (2.84–2.92)	2.91 (2.82–3.01)
**Emphysema/COPD**	1.23 (1.21–1.25)	1.23 (1.21–1.25)	1.23 (1.21–1.25)	1.23 (1.21–1.26)
**Heart disease**	1.17 (1.15–1.18)	1.25 (1.24–1.27)	1.25 (1.24–1.27)	1.25 (1.24–1.27)
**Diabetes**	1.40 (1.38–1.41)	1.33 (1.32–1.35)	1.33 (1.32–1.34)	1.33 (1.31–1.35)
**Cancer**	3.38 (3.33–3.43)	3.49 (3.44–3.54)	3.48 (3.43–3.52)	3.48 (3.42–3.53)
**Stroke**	1.22 (1.19–1.25)	1.27 (1.25–1.29)	1.27 (1.24–1.29)	1.27 (1.24–1.29)
**Alzheimer’s**	0.93 (0.87–1.00)	2.02 (1.95–2.09)	2.00 (1.93–2.07)	2.02 (1.95–2.09)
**Arthritis**	0.80 (0.79–0.80)	0.76 (0.75–0.77)	0.75 (0.74–0.76)	0.75 (0.74–0.76)

## Discussion

Although there are other imputation methods involving machine learning, this study aimed to investigate the effects of four missing data techniques on model coefficients and performance from a linked health survey. Our findings suggest that complete case imputation is not suitable for handling missing data when developing a prediction model. Interestingly, performance measures exhibited minimal changes across mode, single and multiple imputation. However, multiple imputation proved essential in obtaining accurate HRs and confidence intervals for predictors with a higher degree of missingness.

### Complete case

Although complete case imputation is a commonly used technique for handling missing data, it is known to produce bias estimates and large standard errors when the missing data is not Missing Completely At Random (MCAR) [2]. In our study, the prevalence of premature deaths was reduced despite only removing a relatively small amount of the cohort. This observation suggests that individuals who experienced a premature death were more likely to have missing information, indicating a failure of the MCAR assumption. This bias is also particularly evident in the Weibull scale parameter.

While mode, single, and multiple imputation demonstrated only minor variations in the scale values, complete case imputation exhibited noticeable differences. Given that the scale parameter directly impacts the baseline survival, even slight changes can result in differences in predicted probabilities. The Nagelkerke R^2^, c-index and calibration-in-the-large all indicated poorer performance in the models using complete case imputation compared to mode, single, and multiple imputation, both for the female and male models. These results strongly suggest that complete case imputation is an inadequate method and should be avoided [2, 19].

### Comparing performance measures

The results demonstrate similar performance when comparing mode, single, and multiple imputation techniques, with only marginal differences observed. This suggests that for risk prediction, single and multiple imputation offer minimal to no discernable benefit to model performance compared to mode imputation. Furthermore, when examining the calibration plots, all approaches tend to overpredict premature mortality at the higher-risk groups. This is due to less than 2% of the population having a risk greater than 20% risk of a five-year premature mortality. However, the variations between the different imputation methods are relatively minor, suggesting that the choice of imputation method has limited impact on the calibration of the models.

### Comparing hazard ratios and confidence intervals

When comparing the imputation methods, the differences in HRs and confidence intervals are heavily influenced by the percent of missingness in each variable. Variables with less than 1% missingness, such as marital status, self-perceived general health, physical activity, and chronic conditions, show minimal changes in HRs. Multiple imputation, however, tends to yield slightly larger confidence intervals due to the inclusion of additional variance from the HRs across the five imputed datasets. Predictors with a higher degree of missingness, but still below 5%, demonstrate larger changes in HRs and wider ranges in the confidence intervals when employing multiple imputation. These predictors include individual education level, BMI, and smoking status.

Household income surpassed 5% missingness and exhibits notable differences in the female model. Multiple imputation showed the confidence intervals were underestimated in mode and single imputation. While all income quintiles, except the lowest income group (Q1), were found to be statistically significant in mode and single imputation, they were no longer statistically significant when using multiple imputation. For males, household income remained nearly unchanged between mode, single, and multiple imputation, just with larger confidence intervals. Consequently, variables with higher levels of missingness can exhibit unpredictable variations in whether their effects differ across different imputed datasets or remain consistent.

### Limitations

This study should be interpreted considering the following limitations. First, individuals residing in the territories had to be removed given that area-based measures and household income were completely missing. Second, due to convergence issues with multiple imputation, all chronic conditions with low percent missingness were assigned the absence of the given condition (the most common occurrence in the data) and thus the effects of the different imputation methods could not be properly tested for these predictors. The highest missingness of a single variable was less than 11% and thus we could not compare the difference for variables with larger missingness. It is important when encountering data with higher levels of missing to note that the results here may not apply.

## Conclusions

When dealing with missing data in population-based studies, the choice of imputation method depends on the specific goals of the analysis. Researchers should consider the trade-offs between simplicity and accuracy when selecting the appropriate imputation method for their analysis. Both single imputation and multiple imputation are complex imputation methods, requiring more time and methodological knowledge to properly impute missing data. As such, when working with population-based data with similar missingness, if the reader is solely interested in the overall performance of the model and not the individual effects of the predictors, mode imputation is an option. However, if an accurate estimation of predictor effects is of interest, the selection of the imputation method should consider the percentage of missingness in the variables. When predictors have a small percentage of missing values (less than 5%), then mode imputation is satisfactory. Once predictors have a higher percentage of missingness (5% or more), imputed values will introduce greater variability. In such cases, multiple imputation becomes essential to capture the effect of the imputed values accurately.

### Electronic supplementary material

Below is the link to the electronic supplementary material.

Supplementary Material 1

## Data Availability

All data used in this study belongs to Statistics Canada and cannot be shared publicly because of personal health information at the individual level. Data access is only permitted through Statistics Canada Research Data Centres (more information on eligibility and data request process can be found here: https://www.statcan.gc.ca/en/microdata/data-centres).
