# Supplementary material for: The impact of different imputation methods on estimates and model performance: an example using a risk prediction model for premature mortality
Source: Popul Health Metr. 2024 Jun 17;22:13. doi: 10.1186/s12963-024-00331-3 (PMC11181525; doi:10.1186/s12963-024-00331-3)

**Supplementary Materials**

**Figure 3.** Calibration plot of percent of premature deaths and average predicted risk by age group for females.


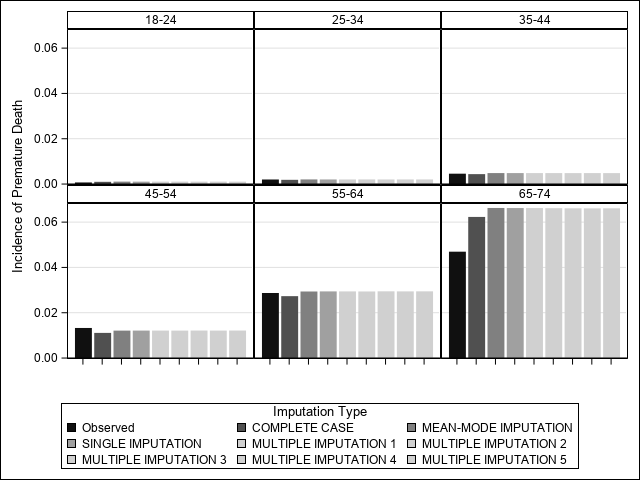


**Figure 4.** Calibration plot of percent of premature deaths and average predicted risk by age group for males.


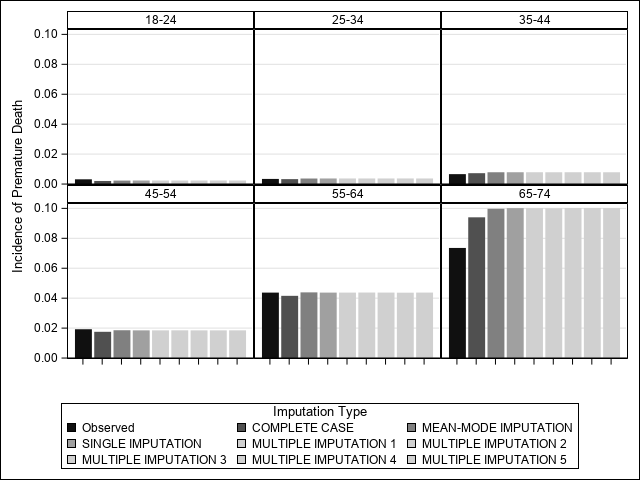


**Figure 5.** Calibration plot of percent of premature deaths and average predicted risk by education level for females.


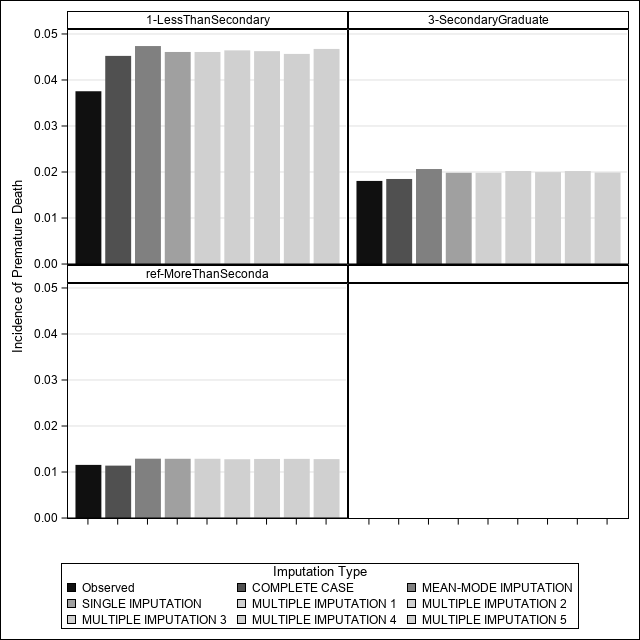


**Figure 6.** Calibration plot of percent of premature deaths and average predicted risk by education level for males.


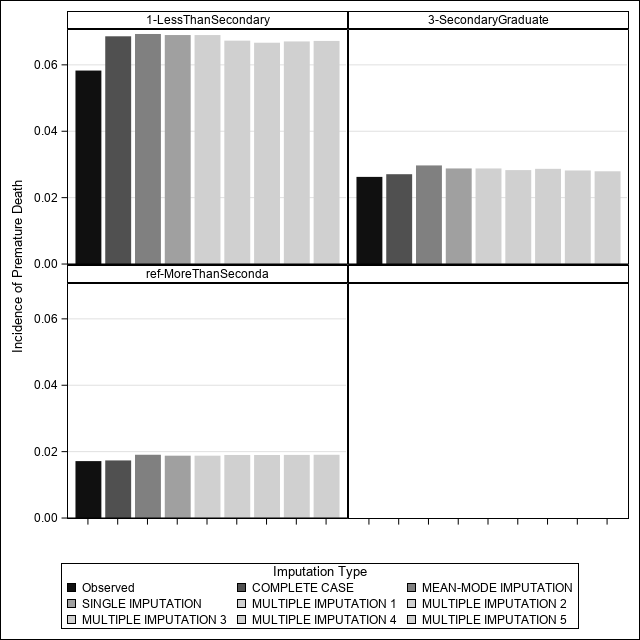


**Figure 7.** Calibration plot of percent of premature deaths and average predicted risk by ethnicity for females.


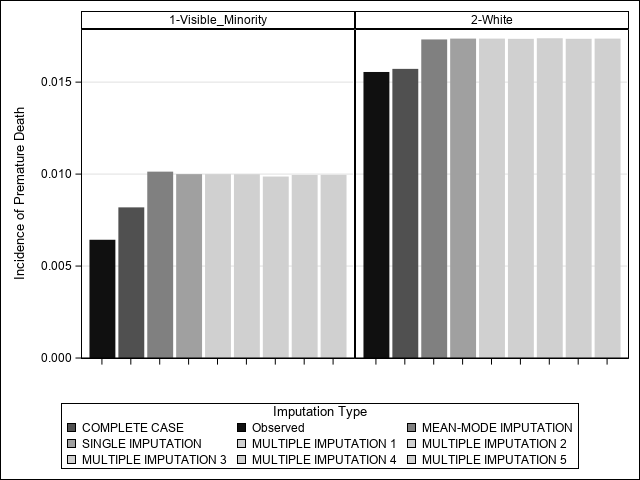


**Figure 8.** Calibration plot of percent of premature deaths and average predicted risk by ethnicity for males.


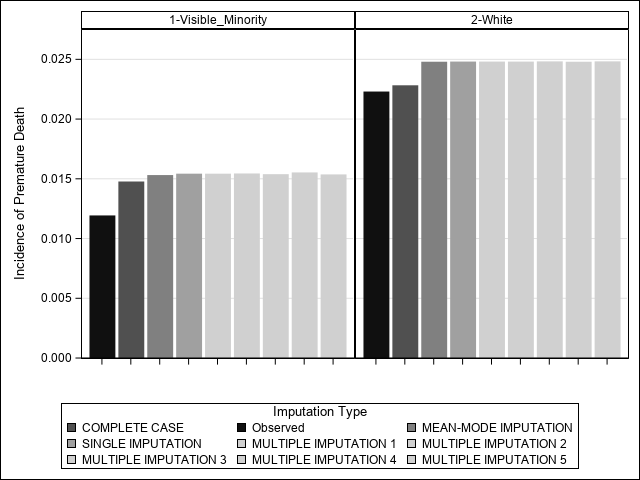


**Figure 9.** Calibration plot of percent of premature deaths and average predicted risk by immigration status for females.


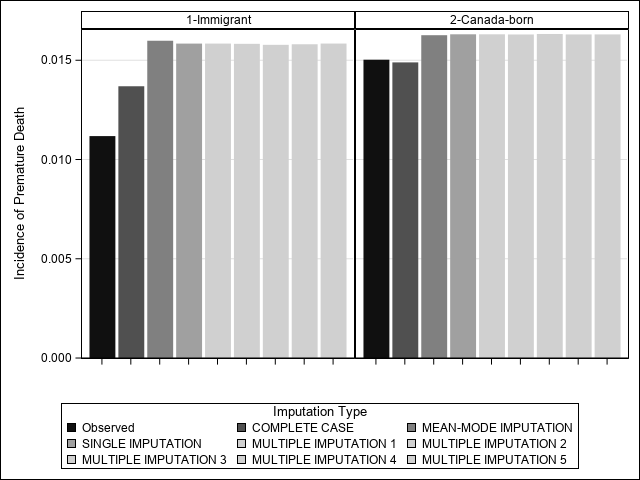


**Figure 10.** Calibration plot of percent of premature deaths and average predicted risk by immigration status for males.


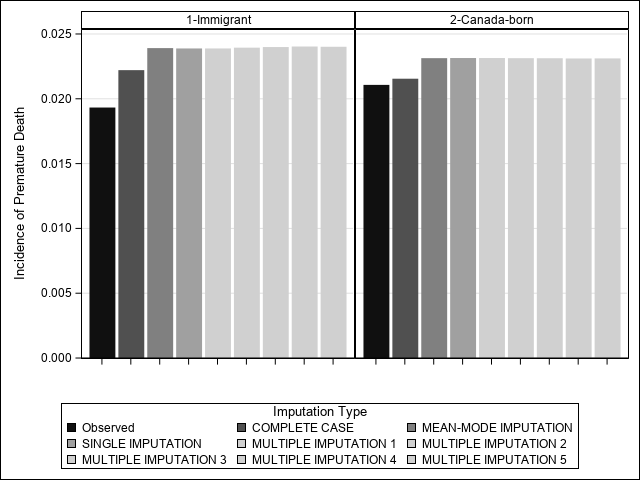


**Figure 11.** Calibration plot of percent of premature deaths and average predicted risk by material deprivation for females.


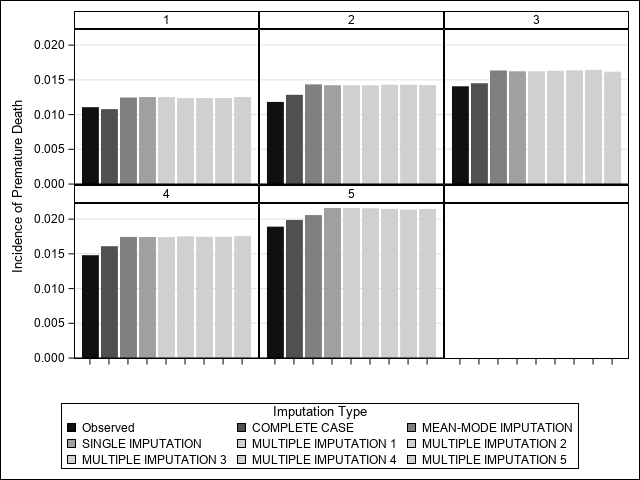


**Figure 12.** Calibration plot of percent of premature deaths and average predicted risk by material deprivation for males.


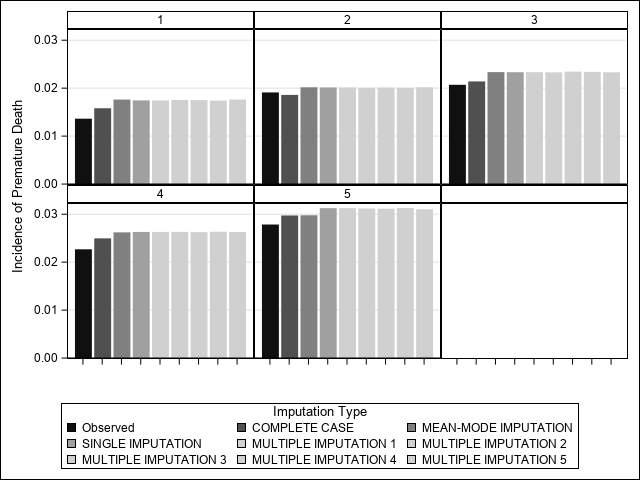

Supplement: Supplementary file 1 — Supplementary Material 1 [file 12963_2024_331_MOESM1_ESM.docx]
